# Zoonotic diseases appeared to be a major hurdle to successful deer farming in Bangladesh

**DOI:** 10.14202/vetworld.2021.2462-2472

**Published:** 2021-09-22

**Authors:** Sajeda Sultana, Nazneen Sultana, Mahmuda Islam, Munmun Pervin, Md. Ariful Islam Khan, Mohammad Abu Hadi Noor Ali Khan

**Affiliations:** 1 Department of Pathology, Faculty of Veterinary Science, Bangladesh Agricultural University, Mymensingh-2202, Bangladesh; 2 Department of Pathology, Faculty of Animal Science and Veterinary Medicine, Sher-E-Bangla Agricultural University, Sher-e-Bangla Nagar, Dhaka 1207, Bangladesh

**Keywords:** deer, enterotoxaemia, mycobacterium, pasteurella, zoonosis

## Abstract

**Background and Aim:**

Due to the diversified lifestyle and fancy ecology associated with Chitra deer (*Axis axis*), deer farming has become popular in Bangladesh. Diseases may be the common constrain of successful deer farming. This study aims to investigate the pathological, bacteriological, and nucleic acid based technologies to identify specific causes of morbidity and mortality of captive deer.

**Materials and Methods:**

Two deer farms and a park deer (designated as farm A, B, and C) entailing 87, 54, and 20 deer, respectively, showed illness and death constitute the study materials. A total of 42 deer died during this investigation. Following death, routine post-mortem examination, histopathology, impression smear staining, isolation, and identification of bacteria were carried out. Polymerase chain reaction (PCR) and reverse transcription PCR were carried out to safeguard the etiology.

**Results:**

Clinically, farm A and B showed the acute phase of illness and park deer showed chronic illness. Case fatality rates were 90%, 92%, and 100% in farms A, B, and C deer, respectively. *Pasteurella multocida* and *Streptococcus pneumoniae* were identified from the visceral organs of farm A deer. Farm B deer was infected with *Clostridium perfringens* type A. Park deer was infected with *Mycobacterium tuberculosis* and hydatid cyst.

**Conclusion:**

The infectivity in farm A deer was due to stress as induced by punishing weather. The infectivity in farm B deer was due to feeding a higher volume of protein in the diet. The park C deer may optate infection from companion man and animals living around. The diseases of captive deer identified mainly were zoonotic. It needs extensive veterinary services and specialized technologies to identify these diseases, monitor the infectivity and eliminate the public health important diseases at early onset.

## Introduction

Deer are widely available in Sundarban mangrove forest and Chattogram hilly areas of Bangladesh. Some people are fond of rearing deer; its skin has been widely used for making shoes, boots, bags, belts, and deer skin is used in esthetic purposes. Wild deer hunting was a common practice in Bangladesh but hunting was legally banned since 2009. The Government of People’s republic of Bangladesh allowed an expansion of forest deer population in the farm, zoo’s, and park condition. Over the decade, it did appear that diseases are the common constraint of successful deer farming. Deer farms are often situated in the vicinity of human shelters and domestic farm animal premises thus poor biosecurity has been maintained in and around them. Therefore, inter-species transmission of pathogens has been considered an important risk factor. Deer has been rearing in a free ranged area in the national park and may come closer with other wild animals. Such deer can play a role as a reservoir for infectious diseases both humans and other animals.

A number of infectious diseases are reported to exist in deer, such as necrobacillosis, coliform septicemia, pasteurellosis, anthrax, clostridial infections, yersiniosis, malignant catarrhal fever, tuberculosis (TB), echinococcosis, endoparasitic infestation, and chronic wasting diseases (CWD). These diseases may cause a high rate of morbidity and mortality, leading to economic losses in captive deer [1-7]. Pasteurellosis in spotted deer has been reported from different parts of the world, including India [8,9]. Sporadic outbreaks of pasteurellosis due to *Pasteurella multocida* have been reported in farmed, park, zoo, and wild deer [10,11]. TB in livestock and farmed deer caused by *Mycobacterium tuberculosis* and *Mycobacterium bovis*, remains a significant economic issue and a public health concern in several developing countries [12]. Due to the existence of a wildlife reservoir of *M. bovis* infection, primarily the Australian brushtail possum, eradicating TB from cattle and farmed deer in New Zealand has proven difficult [13]. The prevalence of gastrointestinal helminthiasis in deer in captivity and wildlife is frequent observation [14,15]. Data are lacking concerning the spectrum of infectious diseases present in deer either in captivity or wild conditions in Bangladesh. The financial impact of a range of clinical and subclinical diseases and mortalities on Chitra deer (*Axis axis*) farms is difficult to assess due to the lack of data on their prevalence or production losses.

Introduction of thoughts for disease diagnosis, control, prevention, and eradication, along with the adoption of that technology may yield significant dividends for successful deer farming [16]. From February 2019 to March 2020, a national park and two regional deer farms (Gazipur and Mymensingh city) of Bangladesh showed huge morbidity and mortality and constituted the study materials.

This study was designed to identify the causes of the morbidity and mortality of deer, with special emphasis on identifying public health important diseases using traditional and nucleic acid-based technologies.

## Materials and Methods

### Ethical approval

This research was approved by the committee of ethical standards, Bangladesh Agricultural University Research System (BAURES) with reference number BAURES/ESRC/2021/Vet/01, dated: 12/01/21.

### Study period and location

On and off-farm investigation of the cases of morbidity and mortality of Chitra deer during the period between February 2019 and March 2020 was carried out. Pathological investigation and laboratory analytical participation were carried out in the Department of Pathology, Bangladesh Agricultural University (BAU), Mymensingh-2202, Bangladesh. For the convenience of study, the deer farms investigated were designated as farms A, B, and C (park deer) according to the date of examining and collecting samples.

### Clinical history

The farms A, B, and C consisting of 87, 54, and 20 deer, respectively. Farm A deer were maintained closer to a forest but were restricted their movement by strong fencing. Farm B deer were maintained in an organized dairy farm composite but there was lacking of strong biosecurity. Farm A and B deer were provided with underground clean water. Farm C deer were allowed to drink pond water but were maintained alone in captivity (park). Farm B deer were maintained with plenty of concentrates supplemented with a higher ratio of protein. During on-farm investigation, the feeding and clinical histories of the affected deer before death were obtained from farm notebook. Close-up clinical inspection was carried out on the deer that appeared moribund following illness. Any discharges noted from the natural orifices of the moribund or dead deer were recorded.

### Pathological examination

On farm, a necropsy was carried out immediately after the death of the deer. Gross changes in the affected system or organs observed were recorded. Representative tissues were collected in fresh falcon tubes, preserved on ice, shifted to the laboratory, and preserved at −20°C for polymerase chain reaction (PCR) detection of suspected illness such as TB, enterotoxaemia (*Clostridium perfringens*), and anthrax. Reverse transcription PCR (RT-PCR) was carried out for the detection of Peste des Petits Ruminanats (PPR) with the samples collected from farm A deer as suspected. Portion of heart, lungs, liver, spleen, kidney, stomach, intestine, trachea, and lymph nodes of dead deer was also collected in 10% buffered neutral formalin for histological examination using hematoxylin and eosin (H&E), gram-staining, and acid-fast staining [17,18]. The paraffin-embedded tissue sections were stained with H&E, gram-staining, and acid-fast stain and mounted using DPX, air-dried and examined at low (10×) and high (40× and 100×) power microscopic fields. The images were captured using a microphotographic system (Cell Bioscience, Alphaimager HP, California, USA) to analyze the observation and documentation of changes.

### Impression smears staining

Impression smears from the lungs, liver, spleen, kidney, and heart blood were made onto clean slides, air-dried and fixed in ice-cold methanol before staining with giemsa [19]. Impression smears onto clean slides were also fixed with transient heat and stained with gram-stain and acid-fast stain [18]. The stained smears were air-dried, examined at 100× microscopic fields and images were captured using a microphotographic system (Cell Bioscience, Alphaimager HP, California, USA).

### Bacteriological examination

The farm A and farm B deer showed acute illness and a higher rate of morbidity and mortality. There was widespread congestion and hemorrhages throughout the body and massive hemorrhages in the intestinal mucosa at necropsy. The necropsy findings suggested that the deer in farm A was suspected of infecting *Pasteurella* spp. or other pneumonia-causing bacteria, including PPR. The farm B deer was suspected to infect with enterotoxaemia or anthrax. Hence, liver, heart blood, intestinal content, and spleen were collected aseptically, processed and attempts were taken to isolate in bacteriological media. The bacteriological media used to identify *Pasteurella* spp. and other species from farm A deer were nutrient broth, and sheep blood agar. Voges–Proskauer (VP) test and sugar fermentation tests were used targeting glucose, sucrose, lactose, mannitol, and maltose to identify species of Pasteurella and Streptococci involved with the disease processes.

For the isolation of *Bacillus anthracis*, the suspected materials were processed and inoculated on sheep blood agar and Polymyxin, lysozyme, EDTA, and thallous acetate (PLET) agar medium [20]. Sulfite polymyxin sulfadiazine (SPS) agar (HiMedia Laboratories Pvt. Ltd. India) media enriched with defibrinated sheep blood was used to isolate *C. perfringens* from farm B deer in anaerobic condition. The SPS agar was prepared by dissolving 4.00 g SPS in 100 mL distilled water, heat to dissolve, and sterilize by autoclaving at 15 lbs pressure (121°C) for 15 min. The medium was cooling down to 50°C, added 7% defibrinated sheep blood and 1 mL (0.05 mg) fungizone (Amphotericin B, Thermo Fisher Scientific INC, NY). Following mixing, the medium was poured onto the sterilized Pyrex test tubes and Petri plates. The test tubes and plates containing SPS medium were allowed to cool, and used in isolation of *C. perfringens* in anaerobic culture. After decontamination of the surface of the visceral organs, a sterile loop was inserted in the visceral organs and streaked onto the Petri plates containing sheep blood agar and SPS. Stab culture was also made in the test tubes containing nutrient agar and SPS medium. Both the aerobic and anaerobic culture was, therefore, incubated in a 37°C incubator. The anaerobic culture was made in a candle jar with apposite sealing and SPS media in test tube were used to isolate *Clostridium* spp. in stab culture. The bacterial inoculation in nutrient broth, nutrient agar, sheep blood agar was carried out in aerobic condition and SPS agar medium in anaerobic condition [7,10,20,21]. The bacteria grown in culture medium were identified by staining the smears onto the clean slides with Gram’s iodine [21] and motility of the bacteria was examined using hanging drop preparation. The species of Pasteurella and Streptococci involved was identified by analyzing colony morphology, using Gram staining of the bacterial smears, and biochemical (glucose, sucrose, lactose, maltose and mannitol fermentation, and VP test) tests [22,23]. The size of the bacteria on stained smears was measured using a digital image capturing software onto the microscope (Carl Zeiss Binocular Microscope, GmbH, Germany).

### Parasitological examination

At the time of necropsy in-depth observation was made to detect parasitic cysts onto the surface and inside the visceral organs. The scolex of the bladder worm was examined under the microscope to detect specific species of bladder worm infestation. Mesenteric veins and gall bladder were examined for the presence of flukes. Gastrointestinal tracts were examined for the detection of tapeworm and nematodes [14]. Fecal content was preserved in 10% buffered neutral formalin, shifted to the Department of Pathology, BAU for the detection of parasitic ova and protozoa (50ül/smear) by direct smear techniques [24].

### PCR and RT-PCR detection of infectious diseases

Aseptically, lungs, liver, kidney, spleen, and intestinal content were collected in cryotubes, snap frozen, and preserved at −20°C. Microbial DNA and RNA from these tissues were extracted using a commercially available DNA extraction kit (Wizard Genomic purification kit, Promega, USA) and RNA extraction kit (SV Total RNA Isolation System, Promega, USA). The purity and concentration of extracted DNA and RNA were measured at 260 nm/280 nm in a Nanodrop™ spectrophotometer (IAEA, Scibersdoff, Vienna, Austria). A 260 nm/280 nm ratio of ~1.8 for DNA and ~2.0 for RNA were considered as “pure” and used for PCR and RT-PCR detection of specific causal agents. The PCR and RT-PCR were carried out using the primer sequences (Table-1) [7,17,25-27] obtained from a commercial source (AIT Biotech, Singapore). The PCR and RT-PCR were carried out in 50 mL reaction volume. The PCR was carried out using GoTaq G2 Green master mix kit (Promega, USA) and RT-PCR using Thermo Scientific Verso 1-Step RT-PCR ReddyMix kit (Thermo Scientific, USA) according to manufacturer instruction. For negative control nuclease-free H_2_O was added instead of template DNA or RNA in the reaction mixture. The reaction was performed in an oil-free thermal cycler (Proplex gradient PCR, USA). The 35 cycles of PCR amplification was carried out using initial denaturation for 4 min at 95°C followed by denaturation at 95°C for 30 s, annealing at 52°C (*PA* gene), 62°C (*16S rRNA* gene), 57°C (*H37RvHP* gene), 55°C (*CPA* gene) for 1.5 min, and extension at 72°C for 2 min. Final elongation was carried out at 72°C for 5 min. The RT-PCR amplification of the selected gene of PPR virus was started with the reverse transcription at 50°C for 15 min. Then the initial denaturation was carried out at 95°C for 2 min followed by 40 cycles of amplification reaction consisting of denaturation at 95°C for 20 s, annealing at 55°C for 30 s, elongation at 72°C for 1 min, and final elongation at 72°C for 5 min. The cDNAs as obtained through PCR and RT-PCR were electrophoresed (WSE-1710Submerge-Mini2322100, China) in 1.5% agarose gel containing ethidium bromide (0.5 mg/mL), and images were captured in a transilluminator (Alpha imager, USA). 100bp DNA ladder (TackIT, Invitrogen, USA) was used in the agarose gels for the evaluation of the size of amplicons.

**Table-1:** Oligonucleotide primers used in PCR and RT-PCR detection of specific causes of illness in farm and park deer.

Genes targeted	Primers name	Sequences (5´-3´)	Organism/Amplicon size	References
*N* gene	PPRNF	gctctgtgattgcggctgagc	PPRV/402bp	Designed
	PPRNR	cctggtcctccagaatcttggcc		
*PA* gene	PA8	gaggtagaaggatatacggt	Anthrax/ 596bp	[25]
	PA5	tcctaacactaacgaagtcg		
*16S rRNA*	TB 1-F3	gaacaatccggagttgacaa	*Mycobacterium tuberculosis* complex/372bp	[26]
	TB 1-R3	agcacgctgtcaatcatgta		
*H37RvHP*	H37RvHPF	gaactcaccgtcggtggtga	*Mycobacterium tuberculosis*/667bp	[17]
	H37RvHPR	ccttgctcgatctctgcgtc		
*MPB83*	MPB83F	cagggatccaccatgttcttagcgggttg	600bp	[27]
	MPB83R	tggcgaattcttactgtgccggggg		
*CPA*	CPAF	gctaatgttactgccgttga	*Clostridium perfringens Type* A/ 324bp	[7]
	CPAR	cctctgatacatcgtgtaagaatc		

## Results

### Symptoms and necropsy findings

**Figure-1:**
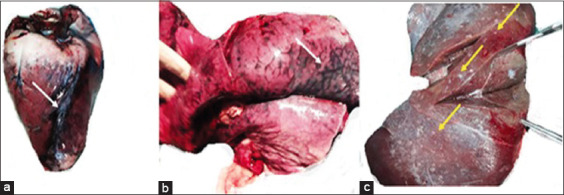
Farm A deer examined at necropsy showed massive hemorrhages in (a) heart (arrow) and (b) lung (arrow). (c) Multiple white necrotic foci were seen on the liver of Farm A deer (arrow).

During this investigation, a total of 29, 13, and 4 deer were infected and 26, 12, and 4 were died in farms A, B, and C, respectively. Clinically deer of farms A and B showed acute phases of illness characterized by high fever, recumbence, diarrhea, reluctant to move, incoordination, and death. Drooling of saliva, nasal discharges and lameness were noted in farm A and B deer. In addition, infected farm B deer showed neurological signs like bending of head and neck with a 90% case fatality rate. Park deer (farm C) showed progressive emaciation and anorexia and infected deer died following a prolonged illness. At necropsy, deer in farms A and B showed widespread congestion and hemorrhages throughout the body. Widespread hemorrhages in the heart (Figure-1a), lobar pneumonia (Figure-1b) and focal coagulative necrosis in the liver (Figure-1c) were characteristics in farm A deer. Farm A deer also showed splenomegaly, hepatomegaly, and hemorrhages in duodenal mucosa.

**Figure-2:**
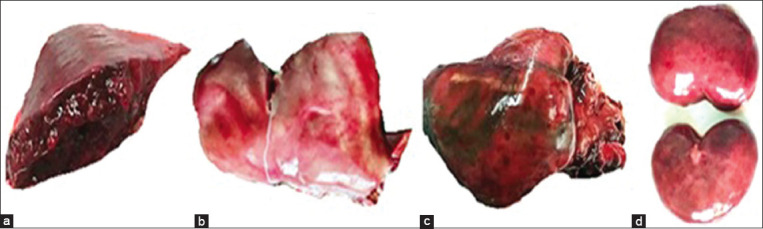
Farm B deer examined at necropsy showed (a) severely congested and consolidated (hepatized) lung. (b) Liver appears degenerated and paler with capillary congestion. (c) Massive congestion and hemorrhages were seen in the myocardium. (d) Multifocal necrosis was seen in the kidneys, and the kidneys were soft, paler, and flabby (pulpy) in appearance.

**Figure-3:**
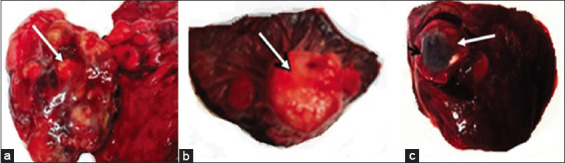
Farm C deer investigated at necropsy showed granulomatous nodules in (a) lung (arrow) and (b) spleen (arrow). (c) Hydatid cyst was seen in the lung of a deer.

Soft, discolored pulpy kidney, necrosed liver, massive enteritis, and unclotted dark color blood were seen in the intestinal lumen of farm B deer. Farm B deer showed hepatized lungs (red hepatization, Figure-2a), degenerated, paler liver (Figure-2b), massive myocardial hemorrhages (Figure-2c), and soft pulpy kidneys (Figure-2d). In farm C deer, the flesh appeared thinner and paler, severely anemic and paler lungs. Multiple nodules were seen onto the lungs (Figure-3a), liver, and spleen (Figure-3b) of park deer. Typical caseous necrosis with cheese-like materials was seen inside the granulomatous nodules. A parasitic cyst (bladder worm) was seen embedded in the lungs of farm C deer (Figure-3c).

### Histopathological observations

**Figure-4:**
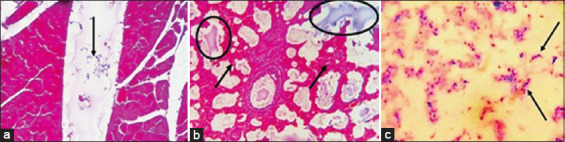
Histopathological examination of (a) heart showed *Streptococcus* spp. (diplococci) in inter-bundular tissues (arrow). (b) There were massive hemorrhages in lung (arrow, 10×) with copious exudate in the alveoli (circle, 10×). (c) Impression smear prepared from the lungs tissues and smears prepared from single colony of agar plate and stained with Gram’s iodine showed *Streptococcus* spp. (arrow).

**Figure-5:**
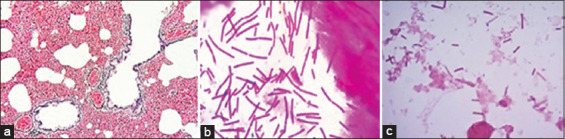
Histopathological examination of (a) lung showed widespread congestion and hemorrhages with emphysema in lungs alveoli (H&E, 10×). (b) Section of heart stained with Gram’s iodine showed rod-shaped bacilli in the myocardium (100×). (c) Impression smears prepared from the kidney and liver of infected and dead deer and stained with Gram’s iodine showed the presence of Gram-positive rod (100×).

**Figure-6:**
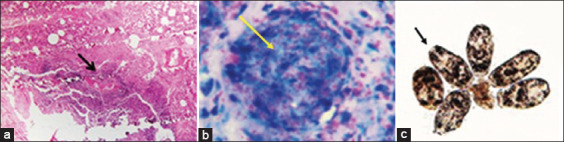
H&E staining of tissue section of (a) lung showed granulomatous reaction (arrow) consisting of inflammatory infiltration of macrophages, lymphocytes and fibroblasts. (b) Lung tissue section stained with acid-fast stain showed pink color bacilli in the granulomatous nodules (yellow arrow). (c) Brood capsule from the cysts of lung was examined under a microscope showed the future heads of the adult tapeworm (protoscolices) within brood capsules.

Severe congestion, hemorrhages, and pneumonic lungs were seen in farms A and B deer. Tracheal mucosa was widely congested, and there was widespread congestion and hemorrhages in the heart and kidney and coagulation necrosis in the liver of farm A deer. Farm A deer were suspected to infect with bacterial diseases and sections of visceral organs were stained with Gram’s iodine showed the presence of Gram-negative *Pasteurella* spp. (bipolar) and Gram-positive *Streptococci* spp. (diplococci) in various organs, including the heart (Figure-4a). Lungs alveoli were distended and showed deposition of pink color exudate (Figure-4b). Widespread congestion, hemorrhages and necrosis were seen in the kidney, spleen, intestine, heart, and lungs of farm B deer. Lungs tissue section stained with H & E showed emphysema, hemorrhages, congestion, and deposition of reactive cells (Figure-5a). Grams staining of tissue sections prepared from the visceral organs of farm B deer showed the presence of rod-shaped Gram-positive bacteria (Figure-5b). Multiple granulomas with dystrophic calcification were seen in the nodular lesions of the lungs, liver and spleen of park deer (Figure-6a). The liver of park deer appeared cirrhotic; there was lymphocytic depletion in lymph nodes and spleen and enlargement of the trabeculae in the spleen. Ziehl-Neelsen staining of the tissue sections of park deer revealed thin rod shape acid-fast bacteria (Figure-6b) in the cytoplasm of epitheloid cells.

### Parasitological investigation

Hydatid cyst was seen in the lungs of farm C deer. Fluid from the cysts was examined under a microscope showed the future heads of the adult tapeworm (protoscolices) within brood capsules. The protoscolices consist of a double row of refractile hooklets (Figure-6c) and 4 round suckers characteristics of the cyst of *Echinococcus granulosus* (hydatidosis). Flukes in the lungs and liver were not seen in any cases. The eggs of *Paraphistomum* spp. and *Haemonchus* spp. were seen in the fecal content at low concentration (1-3 eggs/smear onto the slide) in farms A, B, and C deer.

### Impression smear staining

Blood smears and impression smears prepared from the heart, lungs, liver, and kidney of farm A and farm B deer were stained with Gram’s iodine. Smears from farm A deer showed Gram-negative small rod, bipolar bacteria similar to that of *Pasteurella* spp. and Gram-positive *Streptococci* spp. (Figure-4c). Impression smears prepared from the visceral organs of farm B deer, stained with Gram’s iodine and examined under the microscope showed the presence of Gram-positive rod; the rods were more numerous in kidneys (Figure-5c), liver, and heart. Impression smears prepared from the wall of the granulomatous nodules of park deer, stained with Ziehl-Neelsen staining and examined under the microscope, showed light pink color thin bacteria in the cytoplasm of macrophages.

### Bacteriological isolation and identification

Inoculation of samples from farm A deer showed growth of bacteria in nutrient broth. Bacterial growth from the broth while isolated on sheep blood agar plate showed the growth of both non-hemolytic and hemolytic colonies. The isolated non-hemolytic colonies on blood agar plate were small, mucoid, and dry in appearance. Following Gram’s staining, the bacteria appeared Gram-negative, non-motile, short, and thin rods (0.3-1.0 × 1.0-2.0 mm). The bacteria arranged singly, in pairs, or as short chains and bipolar in nature. The sugar fermentation test revealed that they ferment glucose, sucrose, lactose, and mannitol but did not ferment maltose; the bacteria is VP test –ve, and suggestive for the bacterial species of *P. multocida*. There was also a growth of alpha-hemolytic bacterial colony on blood agar plate (agar under the colony is dark; green hemolysis). The colonies on nutrient agar and blood agar plates were 0.5-2.0 mm in diameter, highly mucoid and glistening. Following Gram’s staining of the smears, the bacteria appeared as Gram-positive cocci, arranged in pairs (*diplococci*), did not form spores, and were non-motile. The bacteria ferment glucose, sucrose, lactose, and maltose but did not ferment mannitol; they are VP test –ve, and characteristic of the bacterial species of *Streptococcus pneumoniae*. Isolated bacteria from farm B deer on SPS agar supplemented with sheep blood and incubated anaerobically showed bacterial growth that formed large, translucent, flat, and filamentous colonies with irregular edges. They produced a double zone of beta hemolysis on SPS agar plate supplemented with sheep blood; the inner zone has complete hemolysis and at outer zone showed partial hemolysis. The bacterial colony smeared on a clean slide and stained with Gram’s iodine showed large rectangular gram-positive bacilli with rounded or truncated ends. The size of the bacteria was about 3-7 mm × 0.4-1.3 mm. The bacteria appear capsulated, containing spore in 5-7% bacteria; they were ­non-motile but showed brawnier movement in hanging drop preparation. The species of the bacteria was suspected as *C. perfringens* and specific species was identified by using published primers in PCR. Bacterial growth, especially *B. anthracis* was not seen in the PLET agar medium incubated aerobically from the farms A and B deer. Farm C deer was suspected to infect with mycobacterium; hence cultural isolation was not attempted.

### PCR and RT-PCR detection of enterotoxaemia, anthrax, TB, and PPR

The genomic DNA was extracted from the isolated bacterial colony on SPS agar, from liver, kidney, and intestine of farm B deer and used in PCR amplified *CPA* gene-specific 324bp fragment of *C. perfringens* type A. The extracted DNA from the visceral organs also used in PCR to identify *PA* gene (596bp) of *B. anthracis* but did not amplify any fragment. Viral RNA was extracted from the lymph nodes and lungs of farm A deer and used in RT-PCR detection of *N* gene of PPR virus but did not amplify any fragment (402bp). The extracted DNA of farm C deer was used in PCR targeting *16S rRNA* gene; amplified 372bp fragment indication the presence of *M. tuberculosis* complex. The extracted DNA from the infected nodules of farm C deer while used in PCR targeting *H37RvHP* and *MPB83* genes, amplified 667bp fragment specific to *H37RvHP* protein gene, and suggested for infectivity due to *M. tuberculosis*. *MPB83* gene-specific fragment was not generated in the PCR.

## Discussion

Chital, cheetal or chitra deer (*A. axis*), also known as spotted deer or axis deer, is a common forest deer in Bangladesh, India, Sri Lanka, Nepal, Bhutan, and in small areas in Pakistan. It is the most common deer species in the mangrove forest (Sundarban) of Bangladesh and India but there were illegal hunting and meat smuggling. With a notion of decreasing unlawful poaching and meat smuggling, Ministry of Fisheries and Livestock and Ministry of Environment and Forests, Bangladesh had decided to legalize the commercial farming of Chitra Deer in 2009. Deer farming business in Bangladesh has an excellent opportunity for earning a healthier livelihood and creating recreation sources. Deer is among the most beautiful wild animals of nature and all types of people are fond of them, especially for their external beauty. The body of Chitra deer is covered with multiple colors and has branching horns, influencing the selection of wild animals in semi-domestication processes. As deer is a wild animal, so people can hardly see them. The deer are available mainly in zoos, where they are confined and displayed to the public. There are dozens of zoos in Bangladesh exhibiting Chitra deer, including Bangladesh National Zoo (Dhaka), Bangabandhu Sheikh Mujib Safari Park (Gazipur), Chittagong Zoo, Comilla Zoo, Dulhazra Safari Park (Coxes Bazer), Gazipur Borendra Park, Khulna Zoo, Nijhum Dhip Park (Noakhali), Rajshahi Zoo, Rangpur Zoo and Mymensingh Zoo. Registered Vet and animal health workers direct monitored the zoos. Besides zoos, some private facilities are also facilitating to rear deer in Bangladesh. The private deer farms have got rare Vet care facilities, and in most cases, the Government Vets have to provide the service for the farm deer.

Nowadays, the global population of Chitra deer is increasing, and people are searching for new farming business ideas that can bloom fancy ecology. As available wild animal species and easy to breed, fed, and manage their biology, the semi-domestication process of Chitra deer is in progress on to the agroecology of Bangladesh. Due to the use of handmade feeds, readily available of visitors close to the deer farms, easy access to domestic and free-living mammals and birds in the farm captivity, farm deer may catch several infectious and parasitic diseases. For domestic deer raising, commercially or as a hobby, the farmer lacked knowledge about the detection and management of diseases of deer. Production of deer gets reduced if they are fallen sick, ultimately the farmer has to suffer very much of management hurdle. Therefore, the disease status of the farm deer was extensively investigated for better management, the safety of the public, vet care facilitators, and eco-health.

Literature available indicated that little study had been carried out on the diseases of farm deer in Bangladesh. A study described the gastrointestinal parasitic infestation of captive and wild deer in Bangladesh; about 76.2% of deer were infected with gastrointestinal helminths; *Paramphistomum* spp. and *Haemonchus* spp. were the most prevalent parasitic infestation in deer [15]. About 69% of gastrointestinal parasitic infection was reported in the wild deer at Char Kukri Mukri of Bhola district, Bangladesh [14]. White tail deer were infected with Lyme disease and hemorrhagic diseases such as epizootic hemorrhagic disease and bluetongue viral disease (BVD) with a higher rate of morbidity and mortality in abroad [28,29]. Anthrax, a septicemic disease, may occur in red deer where it induces edema, hemorrhage, and necrotic lesions with sero-sanguinous fluid exuding from the nostrils [30]. Other potential zoonotic diseases associated with farm or zoo deer include TB and CWD in particular TB, yersiniosis, and malignant catarrhal fever [31,32], brucellosis [33], Q fever, chlamydiosis, leptospirosis, campylobacterosis, salmonellosis, cryptosporidiosis, and giardiasis [3,28]. Diseases of deer are dependent upon the circumstances and ecology where they captured or maintained. Wild deer in captive conditions frequently witnessed visitors, and free-living birds and animals, thus pick up diseases prevailing in those animals, birds, and ecosystems.

This study investigated two deer farms and a park deer in selected areas (Gazipur and Mymensingh) of Bangladesh. The specific causes of infectivity of farm deer were investigated by analyzing clinical records, observing clinical signs, necropsy, histopathology, isolation of bacteria in culture, and PCR. This investigation identified Pasteurellosis and Streptococcal pneumonia in farm A deer, enterotoxemia in farm B deer, and TB in farm C (park) deer. Farm A deer showed acute infection, higher case fatality rate (90%), massive lobar pneumonia, widespread hemorrhages throughout the body, and focal coagulative necrosis on liver. Following isolation of bacteria in culture, *P. multocida* and *S. pneumoniae* were identified. The pasteurella organism grown on blood agar plate was Gram-negative, bipolar in nature and non-hemolytic. Using biochemical test, the bacteria were identified as *P. multocida*. Other bacteria grown on blood agar media was alpha-hemolytic, which was characterized by the greenish appearance of the blood agar under the colony. Alpha hemolytic species cause oxidization of iron in hemoglobin molecules within red blood cells, producing green hemolysis on blood agar [34]. Following Gram staining, the bacteria appeared as diplococci and Gram-positive. The Gram-positive, diplococci pathogens comprised *S. pneumoniae* and some species of enterococcus bacteria. Results of alpha hemolysis on sheep blood agar, sugar fermentation tests and VP tests indicated that the bacterial species contained the characteristics of *S. pneumoniae*. Species of *S. pneumoniae* are mostly a pathogen of human respiratory tract and opportunist to hypoimmunity, conferring challenges to the clinicians to attain a diagnosis [35].

People with the pneumococcal disease can spread the bacteria to others when they cough or sneeze. The farm deer may catch the organism from the people served for the farm or from the visitors. On the other hand, the diplococci bacteria enterococcus is involved with enterococcal meningitis, an uncommon nosocomial disease of human. The commonly occurring enterococcus is *Enterococcus faecalis*. Colonies of *E. faecalis* on sheep blood agar in aerobic atmosphere typically exhibits gamma-hemolysis (non-hemolytic), considered a differential point to identify *S. pneumoniae* from *E. faecalis*. The diplococcal bacteria isolated in this study were *S. pneumoniae*. This is for the 1^st^ time in Bangladesh detected diplococcal bacterial infection in farm deer. Literature available indicated that diplococcal infection in deer is not a regular illness. Hemorrhagic septicemia is common in farm animals but not frequently reported in deer [8-11,36,37]. As there was profuse nasal discharges, diarrhea, and a higher rate of morbidity and mortality, a case of PPR was also suspected.

RT-CPR test was carried out targeting *N* gene of PPRV but did not amplify any fragment of the gene and the deer was not infected with PPR. PPR is endemic in small ruminants of Bangladesh [38,39], ruminants may catch the infection but the farm A deer was not infected with PPR. The disease outbreak occurred in farm A deer in April 2019; at that time, the environmental temperature was higher with high humidity and stormy weather. These all could have posed stresses onto the deer; stresses and lack of vaccination against pasteurellosis might have predisposed farm A deer to *P. multocida* and *S. pneumoniae* infections. Deer may pick up *S. pneumonia*e infection from humans, as a zoonotic pathogen, its importance is laying with the causation of pneumonia in humans. The deer may act as a carrier of the infection and may disseminate infection to the vet caretaker, farm caretakers or visitors.

Out of 54 deer in farm B, 13 were infected and 12 were died within 48 h of ailment. Due to moderately high fever (102°-103°F) the deer went off feed, the infected deer appeared lethargic, showed blackish diarrhea, and finally, death was onset following 12-24 h of illness. Neurological symptoms such as head and neck extended back over their withers was seen. There was a history of feeding concentrate containing a higher amount of protein. At necropsy, soft pulpy kidney, fragile liver, and enteritis was seen. Following impression smear staining with Grams iodine, Gram-negative rod shape bacteria was seen in the heart, lungs, liver, kidney, and spleen and the case was suspected as enterotoxaemia or anthrax. Extracts from visceral organs were incubated on SPS medium to isolate clostridium bacteria in anaerobic conditions and Gram-positive rod-shaped bacteria was isolated in culture. The extracts from the visceral organs, while cultured on blood agar plate in aerobic condition, did not show growth of any rod-shaped bacteria. As there was plenty of Gram-positive rod in the tissues and suspected as anthrax, the extracted DNA from the tissues was used in PCR detection of pXO1 (*PA* gene, 25) of *B. anthracis*. None of the samples generated *pX01* gene-specific amplicon in PCR and the farm deer was, therefore, not infected with *B. anthracis*.

The species of the bacteria as isolated in anaerobic culture from the visceral organs and Gram-positive rod-shaped bacteria as seen in tissues following Gram staining of the sections the diseases was suspected most likely due to *C. perfringens*. The extracted DNA from the isolated colonies and from the visceral organs was used in PCR, amplified 324bp fragments specific to *CPA* gene of *C. perfringens* type A. The farm B deer was, therefore, infected with *C. perfringens* type A bacterium. Application of balanced diet with optimum protein supplementation and oral supplementation of Ciprofloxacin plus antipyretic drugs prevent further relapse of the cases. The case was, therefore, diagnosed as enterotoxaemia. Previously enterotoxaemia in deer was reported with an acute phase of illness; clinically enterotoxaemia progresses so quickly, animals may have succumbed to death without erstwhile signs of disease [1,5,7].

The farm C deer (park deer) was suffering from chronic and emaciating diseases and out of 20 deer in the park, four died during the investigation period. Before death, the deer appeared cachectic and recumbent with respiratory distress. At necropsy, numerous nodular growth was seen in the visceral organs and the case was suspected as TB. The sections of the nodular lesions and impression smears on the clean slides were stained with Ziehl-Neelsen stain and acid-fast bacilli were seen in the cytoplasm of macrophages. PCR was carried out using the extracted DNA from the lung lesions found to yield 372bp and 667bp amplicons suggestive for the infectivity due to mycobacterium TB complex and *M. tuberculosis*, respectively. Hydatid cyst was also seen in the lungs of a dead deer. The deer in farm C were, therefore, infected with *M. tuberculosis* and hydatidosis, important pathogens having public health significance. Since the emergence of global deer farming as an alternative farming enterprise over the past 30 years, there has been an increasing awareness of the potential threat posed by TB.

TB, caused by *M. bovis* has been found in deer in every country involved with deer farming [40]. Bovine TB due to *M. bovis* is quite rare and account for less than 2% of total TB, or TB cases in the USA [41]. Three commercial deer herds in New Zealand, each contain more than 500 deer, experienced outbreaks of TB ranging from 2% to 6% prevalence. Detection of TB in live deer is of major hurdle and therefore, TB remained silent in farm or park deer. Reports are scanty in Bangladesh describing the occurrence of TB in deer due to *M. tuberculosis*. TB due to *M. tuberculosis* in deer is expected to derive from human. Therefore, human deer interaction was assumed to play a role in disseminating TB in park deer but detection of TB in captive deer is a bit more difficult until the deer showed progressive emaciation or ill health.

Mid cervical skin tests are sometimes designated as the primary test and a comparative cervical test as an ancillary test. In an attempt to enhance TB eradication, ancillary blood tests comprising; lymphocyte transformation tests (LT); and enzyme-linked immunosorbent assays (ELISA) were used [42]. Virtually controlling of farm deer and use of intradermal tuberculin test is impractical. Deer require to capture using a dart gun or tranquilizer gun before injecting tuberculin. As deer is a sensitive wild animal, controlling procedure is stressful to the deer; results in self-trauma or iatrogenic injury during restraint and struggling and, even under ideal circumstances, injury and mortality may occur at the time of controlling [43]. To perform tuberculin tests, it requires to control the deer twice, firstly at the time of injecting tuberculin and secondly measurement of skin reaction following 48 to 72 h of injection. Moreover, performing LT test and ELISA also requires to collect blood and to control animals. Therefore, detection of TB in farm deer is seldom practice due to the limitation of control procedure. Therefore, this study was examined dead animals to identify the specific cause of TB using necropsy, impression smear staining, histopathology, and PCR technologies.

TB in farm, zoo, or park deer is a frequently reported or under-reported condition and the deer mostly earn the sickness from caretakers or invader animals. Geographically, most human TB cases in 2018 were reported in South-East Asia (44%), Africa (24%), and the Western Pacific (18%). Smaller percentages of human TB are reported in the Eastern Mediterranean (8%), the Americas (3%), and Europe (3%). A total of 1.5 million people died from TB in 2018. Eight countries accounted for two-thirds of the global total human TB [44]; India (27%), China (9%), Indonesia (8%), the Philippines (6%), Pakistan (6%), Nigeria (4%), Bangladesh (4%), and South Africa (3%) account for the highest total. Bangladesh in the WHO’s list among the global top 10 high TB burden county and mostly caused by *M. tuberculosis* and rarely by *M. bovis*. *M. bovis* and *M. tuberculosis* are now predominantly occupational zoonosis with potential risk for workers on farm, in abattoirs, and in Zoos of developing countries [45,46]. The park C deer was maintained in free-range system and had good contact with squirrels, park workers, dogs, cats, crows, etc. These animals, birds, and caretakers may have transmitted TB to park deer or they may share it. Transmission of TB in cattle and farmed deer in New Zealand was greatly influenced by the existence of a wildlife reservoir of *M. bovis* infection, principally the Australian brushtail possum [13]. The principal source of TB in park deer was not confirmed in this study but the increased incidence of TB due to *M. tuberculosis* in this park indicated that external sources were prevailing and needed to uncover the clues.

Pulmonary echinococcosis (hydatidosis) was found in the lungs of a park deer. Wild and domestic animals such as elk, deer, cattle, sheep, and goats usually act as intermediate host of *E. granulosus* [47]. Globally echinococcosis is a zoonotic larval infection of the tapeworm (cestode) *E. granulosus* [48]. The definitive hosts of the cestode are carnivores and most notably, dogs and cats. Other intermediate hosts ingest eggs or gravid proglottids that are excreted in the definitive host’s feces, causing the infection as hydatidosis in humans, cattle, sheep, goats and other herbivores [49]. A human being carrying hydatid cyst may be the end-stage intermediate host as there is no chance of definitive host’s (carnivores) to consume cystic stage of the worm from human body. However, the incidence of such disease is different in various parts of the world, including Europe. The roe deer is a Eurasian wild cervid and is infected with various diseases. The antibodies detected against Pestivirus (1.5%), Herpesvirus (0.2%) and *M. avium paratuberculosis* (9.2%). MAP antibodies were detected in seven of the eight populations [50]. CWD is enzootic in some areas in North America. The CWD is fatal and caused by contagious prion disease. The disease has been reported in deer, elk and moose in the USA and Canada, and in South Korea following the importation of infected animals. Cases of CWD were reported in Europe, in a Norwegian free-ranging reindeer in Southern Norway [51]. The important bacterial disease affecting deer in UK includes para TB (Mycobacterium avium subsp. paratuberculosis), bovine TB (*M. bovis*), pseudotuberculosis (*Yersinia pseudotuberculosis*), and colibacillosis *(Escherichia coli* O157). The viral diseases affecting farm deer in UK included foot and mouth diseases (FMD) virus, luping-ill virus, west Nile virus, bluetongue virus, and bovine viral diarrhea virus [52]. The disease distribution pattern in Europe and in Bangladesh appeared a bit different due to the different ecology of the farms, management systems introduced and period of time investigated.

### Follow-up study

Two deer farms and a park deer investigated were strongly monitored during the investigation period. Farm owners were suggested not to introduce any deer from other sources. As the Chitra deer breed quickly, the farm A and B deer were found to have their sound reproductive behavior, gave birth once in a year and produced 1-2 kids per doe as expected. The farm deer were vaccinated against FMD and PPR as the diseases are endemic in Bangladesh in small ruminants. Farm B deer were maintained with a balanced ration and suggested to be fed both concentrated and roughages. Following 6 months of this investigation, a park deer was fallen sick, was suspected to infect with TB, sacrificed, the deer were infected with *M. tuberculosis* as well. Morbidity in two deer farms was not noted following 6 months of this investigation.

## Conclusion

To identify causes of mortality of captive deer, a total of 42 dead deers in three captive conditions were extensively investigated; acute infectivity was seen in two deer farms and chronic infectivity in a park deer. The main health issues in farm A deer were pasteurellosis and were due to sudden stress imposed by punishing weather. The farm B deer were infected with enterotoxaemia, the farm deer was maintained on concentrate supplemented with a higher level of protein, that have created anaerobic condition in the gut and predispose deer to clostridial infection. The park C deer was infected with *M. tuberculosis* and hydatidosis. The diseases identified in farm and park deer such as TB, hydatidosis, streptococcal pneumonia, and enterotoxemia are all zoonotic. The diseases identified were typically influenced by faulty management, non-vaccination against pasteurellosis and TB, the introduction of stress, inefficiencies of providing a balanced diet and accessibility of free-living animals and birds. It needs standard vet care facilities in all the farms and park deer, regular training of farm owners and park managerial teams to maintain healthy ecology in the farm and park. There are hundreds of farms and parks in Bangladesh rearing chitra deer but the deer carrying public health important diseases is largely ignored. There are limited Vet care faculties to serve the deer. There are government veterinary field disease investigation laboratories in each division of Bangladesh, but diseases of wild animals seldom reported to this laboratory, thus the diseases of deer are largely ignored and silently may be a source of infection to man and animals living around. Early detection of this infectivity is required, which may provide better health management of captive deer and livelihood of the people living around them.

## Authors’ Contributions

SS, MP, and MAHNAK: Designed the study, generated a research fund, and tuned the manuscript for submission. SS, NS, MI, and MAIK: Helped in collecting and processing samples and did PCR, RT-PCR, and gel electrophoresis. SS and MP: Carried out the bacteriological analysis. SS, MP, and MAHNAK: Drafted and revised the final manuscript. All authors read and approved the final submission.
